# In defence of a single body of clinical and public health, medical ethics

**DOI:** 10.1186/s12913-020-05887-y

**Published:** 2020-12-09

**Authors:** Jean-Pierre Unger, Ingrid Morales, Pierre De Paepe, Michel Roland

**Affiliations:** 1 Department of Public Health, Institute of Tropical Medicine, Nationalestraat 155, B-2000 Antwerp, Belgium; 2 Office de la Naissance et de l’Enfance, French Community of Belgium, Chaussée de Charleroi 95, B-1060 Brussels, Belgium; 3 Département de Médecine Générale, Université Libre de Bruxelles, Route de Lennik, 808, BP 612/1, B-1070 Brussels, Belgium

**Keywords:** Medical ethics, Public health ethics, Disease control, Clinical integration, Doctor-patient relationship

## Abstract

**Background:**

Since some form of dual clinical/public health practice is desirable, this paper explains why their ethics should be combined to influence medical practice and explores a way to achieve that.

**Main text:**

In our attempt to merge clinical and public health ethics, we empirically compared the individual and collective health consequences of two illustrative lists of medical and public health ethical tenets and discussed their reciprocal relevance to praxis.

The studied codes share four principles, namely, 1. respect for individual/collective rights and the patient’s autonomy; 2. cultural respect and treatment that upholds the patient’s dignity; 3. honestly informed consent; and 4. confidentiality of information. However, they also shed light on the strengths and deficiencies of each other’s tenets. Designing a combined clinical and public health code requires

- fleshing out three similar principles, namely, beneficence, medical and public health engagement in favour of health equality, and community and individual participation; and
- adopting three stand-alone principles, namely, professional excellence, non-maleficence, and scientific excellence.

Finally, we suggest that eco-biopsychosocial and patient-centred care delivery and dual clinical/public health practice should become a doctor’s moral obligation.

We propose to call ethics based on non-maleficence, beneficence, autonomy, and justice – the values upon which, according to Pellegrino and Thomasma, the others are grounded and that physicians and ethicists use to resolve ethical dilemmas – “neo-Hippocratic”. The neo- prefix is justified by the adjunct of a distributive dimension (justice) to traditional Hippocratic ethics.

**Conclusion:**

Ethical codes ought to be constantly updated. The above values do not escape the rule. We have formulated them to feed discussions in health services and medical associations. Not only are these values fragmentary and in progress, but they have no universal ambition: they are applicable to the dilemmas of modern Western medicine only, not Ayurvedic or Shamanic medicine, because each professional culture has its own philosophical rationale.

Efforts to combine clinical and public health ethics whilst resolving medical dilemmas can reasonably be expected to call upon the physician’s professional identity because they are intellectual challenges to be associated with case management.

## Background

Medicine has had moral objectives alongside its technical purpose since Hippocrates. Today, the doctor’s internalisation of professional values (principles guiding action) remains essential to medical practice because ethics is a motivational driver of care quality and both “regulation-and-control” systems (as in European “corporatist” healthcare states) and “command-and-control” systems (as in HMOs) are relatively ineffective in securing most patients’ rights: both have shown their limits in curbing unethical practice in industrial and low and middle-income countries (LMICs) [1]. Physicians should thus assume ethical values as a sine qua non for their exercise of medicine. The internalisation of values is in turn favoured by the existence of clear though possibly contradictory principles, by clinical debates to reflect on the tensions between these values, and by a moral community that safeguards the dissemination of values amongst its members.

Whilst ethics is key to the profession, medical practice often reduces access to care and even causes health problems of demographic and epidemiological importance [2], and the contemporary political economy of care does not help matters. The increasing commoditisation of care, privatisation of health insurance, and tendency of medical culture to become materialistic are driving physicians to take self-serving clinical and public health decisions. Commercial health insurance schemes, for-profit hospitals, and disease control organisations tend to

- limit the physicians’ autonomy. For instance, the U.S. health industry enforced managed care with 1.5 million white-collar workers busy limiting access to care and imposing clinical conduct on barely half as many doctors [3]. And ahead of privatisation, public services also hobbled professional autonomy to control public expenditures, under the pretexts of evidence-based medicine (EBM) [4], quest for efficiency [5], and good governance [6].
- promote clinical guidelines that ignore trade-offs (effectiveness vs. efficiency; patient’s security vs. autonomy; etc.) that must be agreed upon by doctor and patient: T. Greenhalgh summarised the ethical difficulties with EBM as follows: “The evidence-based ‘quality mark’ has been misappropriated by vested interests, …, inflexible rules and technology-driven prompts may produce care that is management driven rather than patient centred” [4];
- reduce the physician’s right to oppose regulations on moral grounds (the “right” of heteronomy) [7];
- lobby to weaken the government’s regulatory capacity in the U.S. [8] and in LMICs [1, 2, 9]; and
- rely inordinately on material incentives to motivate physicians. For example, managed care techniques associate income with clinical decision making and thereby stimulate commercial medical practice and a medical culture of self-interest [10, 11].
- In addition, pharmaceutical companies distribute gifts to clinicians [10] and give salary top-ups, for instance in international disease control programmes (Global Health Initiatives).

This context is of interest here not for the strategies it could justify to improve the ethics of physicians but for the characteristics that codes of conduct for physicians must acquire in order to limit the harmful impact of materialistic culture and commercial health care financing and management on medical practices.

The twentieth century spawned a significant development in Hippocratic ethics. Since WWII, the interference of a third party (social security and commercial insurance companies) in the doctor/patient relationship has required the physicians’ code of conduct to become distributive and to address access to and rationing of health care on the ground of the common good [12]. In the conceptual sphere, professional values have opened up to medical virtues. In 1993, Pellegrino and Thomasma discussed the importance of the physician’s professional virtues for their practical reliance on ethical values [13] 1.

Medical ethics and culture must now also adapt to progress in artificial intelligence so that medical practice can use AI most gainfully. Furthermore, physicians should give public health content to their clinical practice and clinical content to their public health practice so as to maximise their impact on collective health whilst individualising care delivery and so be in line with eco-biopsychosocial^2^ and patient-centred care concerns. To do so, doctors can build and lead teams; reflect on practice; educate, coach, and train; improve the organisation of their health services; coordinate and evaluate healthcare; contribute to disease and health risk control; do operational research; and lobby health policies.

When physicians practice and connect these activities, they could be called “manager physicians”, although managers are traditionally defined as persons entrusted with decision-making aimed at achieving their institutions’ predetermined goals. Any doctor can adopt a manager-physician role without necessarily being commissioned as such by her/his institution.

Historically, public health and medicine have been operationally and epistemologically divorced. Public health was generally defined as the science of collective care and sanitation. It addressed the control of biological and environmental, and, to a lesser extent, social and cultural health determinants by means of prevention, promotion, and standardised curative care programmes. In contrast, clinical medicine was seen as the science of individual, “discretionary” curative care. Such definitions reflected a parallel divorce between curative and preventive care practices, with prevention generally taking the form of government-planned collective health programmes (possibly delivered by individual physicians) and individual care being initiated at the patient’s request and delivered by private entities or self-employed physicians (in Bismarkian systems such as in the French or German health systems) or by public services (in Beveridgean systems such as in the UK’s National Health Service or in the Spanish *Sanidad*).

These divisions have, however, proved inaccurate because mass prevention programmes, from immunisation to sexual education, account for a small portion of available prevention and promotion: both should generally be custom-tailored to individual and family characteristics and thus be integrated into curative care service delivery. In High Income Countries (HICs), for instance, public health programmes address quite a broad array of conditions that require custom-tailored health risk management, e.g., obesity, smoking, drug use, dropping out of school, language development of infants, domestic violence, psychomotor development, and metabolic disease detection programmes. If these health care-dependent interventions are to be effective, they need, as a rule, to be integrated in health services where care is acceptable and accessible and material conditions (consultation time, staff workload, etc.) permit it [14]. This is far from common practice. For instance, antenatal and under-five clinics in Belgium are by and large entrusted to a general health prevention organisation, the “Birth and Child Office”. In LMICs, services are much more segmented and fragmented. The Congolese health system, with its fifty-seven different (internationally-financed) disease-specific programmes, is, like most others in Sub-Saharan Africa, so plagued by bureaucratic red tape that its health services often cannot offer any decent-quality care.

If some dual clinical/public health, medical practice is desirable, the epistemic borders between medical and public health knowledge should be eliminated. Earlier in the series we therefore advocated [15] merging public health and medical knowledge into a single health science to enlighten professional, socially-motivated medical practice. In this article we discuss why and how the ethics of medicine and public health should similarly be combined to guide the proposed integration of medical and public health practices and to provide a common language for clinicians and public health practitioners.

We propose to call ethics based on non-maleficence, beneficence, autonomy, and justice – the values upon which, according to Pellegrino and Thomasma [13], the others are grounded and that physicians and ethicists use to resolve ethical dilemmas – “neo-Hippocratic ethics”. The “neo” prefix is justified by the adjunct of a distributive dimension (justice) to traditional Hippocratic ethics.

## Main text

To lay the groundwork for a proposed combined code of clinical/public health ethics to guide clinical, public health, and dual practices, we compared the tenets of two influential medical and public health ethical principles, the elements of which we deemed to be illustrative of the two fields, to come up with a single code of principles.

The medical principles were those formulated in 1994 in the BMJ [16]. They are derived in part from the Hippocratic codex. For the sake of clarity, we have numbered these tenets M1-M8:

- M1. Patients’ autonomy should be respected (“respect”).
- M2 Patients have the right to refuse or choose their treatment (“respect” supposes “informed consent”).
- M3 The patient (and the person treating the patient) has the right to be treated with dignity (“patient’s dignity”).
- M4 Practitioners should act in the patient’s best interest (“beneficence”).
- M5 First, do no harm” (“non-maleficence”).
- M6 In treatment and diagnosis, practitioners should aim to distribute scarce health resources according to need (“fairness” or “justice”).
- M7 Patients’ consent should be informed by the prevailing evidence. “Honestly informed consent” assumes that doctors should not take advantage of the information asymmetry.
- M8 Information on patients should be treated confidentially (“medical secrecy”).

Since these medical tenets are derived from patient’s rights, they may be viewed as essentially clinical tenets.

Regarding public health ethical principles, we chose the tenets of the Public Health Leadership Society [17], numbering them PH(a) to PH(l).

- PHa Public health should address principally the fundamental causes of disease and requirements for health with the aim of preventing adverse health outcomes (aetiological approach).
- PHb Public health should achieve community health in a way that respects the rights of individuals in the community (respect for individual rights).
- PHc Public health policies, programmes, and priorities should be developed and evaluated through processes that ensure an opportunity for input from community members (democracy).
- PHd Public health should advocate and work for the empowerment of disenfranchised community members with the aim of ensuring that the basic resources and conditions necessary for health are accessible to all (equity).
- PHe Public health should seek the information needed to implement effective policies and programmes that protect and promote health (scientific excellence).
- PHf Public health institutions should provide communities with the information they have that is needed for decisions on policies or programmes and should obtain the community’s consent for their implementation (community participation).
- PHg Public health institutions should act in a timely manner on the information they have within the resources and the mandate given to them by the public (effectiveness within a mandate).
- PHh Public health programmes and policies should incorporate a variety of approaches that anticipate and respect diverse values, beliefs, and cultures in the community (cultural respect).
- Phi Public health programmes and policies should be implemented in a manner that most enhances the physical and social environment (health promotion in the environment).
- PHj Public health institutions should protect the confidentiality of information that could harm an individual or community if made public. Exceptions must be justified on the basis of the high likelihood of significant harm to the individual or others (confidentiality of information).
- PHk Public health institutions should ensure the professional competence of their employees (professional excellence).
- PHl Public health institutions and their employees should engage in collaborations and affiliations in ways that build the public’s trust and the institution’s effectiveness (public health activism).

We matched these two lists and classified the ethical tenets as being shared, incompletely equivalent, stand-alone (missing from one of the lists), missing from both lists, and not a principle. To identify principles that might have been missing from both lists, we derived professionals’ duties from patients’ and communities’ healthcare rights conceived of as per the WONCA quality-of-care criteria for general practice/family medicine [18]. To see their relevance to both medical and public health practice, we used illustrations drawn from the experience and knowledge gained by the authors’ teams.

In the event of contradictions between the clinical and public health ethics, we decided to give primacy to clinical tenets over public health ones, preferring clinical to public health terminology and clinical to public health concepts. This decision is value-based and mainly operational due to:

- the authors’ fidelity to the medical, ethical tradition; and
- the quest for disease control effectiveness. Disease control programmes that entail clinical interventions (that is, the vast majority of them) are not acceptable to people unless their interventions are delivered “with care”: clinical quality-of-care criteria thus ought to apply as well to public health programmes in all stages (planning, implementation, and evaluation).

The result of the comparative analysis consists of a non-exhaustive series of values set out in the subtitles of this section. They are presented to contribute to the development of ethical principles and new jurisprudence as well as systematically to enrich clinical case discussions and presentations with ethical inquiry and reflection.

### Physicians should act in the patient’s and community’s best interests (“beneficence”) (M4)

The beneficence principle appears in the medical list only. It should apply to public health as well. Acting in a patient or community’s interest is similarly incompatible with an ulterior commercial or institutional motive such as maximising profits. For instance, just as family doctors and pharmacists should keep patients and drugs separate, community medicine and public health doctors should apply public health goals whilst being extremely demanding as regards the scientific aspects of their choice of prevention (based on, for example, the efficiency of vaccination) and of potential environmental harm (when, for example, protecting children in kindergartens in rich countries from the abusive use of chemical hygiene products and disinfectants).

Whilst Beauchamp and Childress regarded beneficence as the physician’s primary obligation that unifies the theory of medical ethics [19], Tassano questioned the idea that beneficence might in some cases have priority over autonomy, arguing that violations of autonomy more often reflect the interests of the state or health professionals (the medicalisation of psychosocial complaints, for instance) than those of the patient [20]. “Today,” Pellegrino and Thomasma conclude, “non-maleficence, beneficence, autonomy, and justice have become the reference tetrad par excellence that physicians and ethicists use to resolve ethical dilemmas “ [13].

The acquisition of a distributive dimension is undoubtedly one of the most significant advances made in Hippocratic ethics, because a social justice mission is now attached to the medical profession. Accordingly, doctors are required to consider the distribution of their working time, use of the medical equipment they manage (such as ventilators in treating Covid-19 patients), and prescription of drugs paid reimbursed by the social security from an ethical angle. The conceptual and practical importance of this change permits to term “neo-Hippocratic”, the contemporary medicine that is practised as an “ethical end-in-itself” and, in particular, abides by a distributive justice.

### Physicians should respect and promote individual and community autonomy in health (M1)

The medical codes that we studied provide for respecting the patient’s autonomy (M1). Acting for the good of the patient or community without their participation is simply not tenable, because rights holder compliance is needed. That is even more the case when living conditions are at stake. However, not only does this tenet give the patient the right to choose her/his treatment, but it also imposes on the doctor the obligation to reinforce the patient’s independence from the disease and its medical solutions. For example, endocrinologists need to teach patients how to control their diabetes with insulin depending on their skills and type of disease.

Similarly, public health programmes can hinder a community’s health autonomy when, for example, they organise networks of community health workers to tackle health problems, say, common diarrhoeas or fevers, that the community effectively resolves without them. Africa is rife with such examples.

Like clinicians with patients, public health physicians must strengthen the autonomy of communities. Community autonomy in health requires the existence of mechanisms for mutual support and the exchange of experiences and information about the social, cultural, and economic determinants of health (as do Alcoholics Anonymous and other therapeutic groups). Doctors should, therefore, favour the development of such community dialogue processes and mechanisms.

### Clinicians and public health physicians should provide eco-biopsychosocial care

Neither of the two lists that we studied discusses this value. If we accept that biopsychosocial care is a patient’s right, we ought to agree that the doctor has an obligation to deliver care that connects the environmental, biological, social, and psychological dimensions of diagnosis, treatment, and prevention. However, such care ought not to be given to the detriment of the patient’s autonomy, for example, with undue medicalisation of psychosocial complaints. Just like clinicians, public health physicians ought to aim to deliver eco-biopsychosocial care in community medicine practice and disease/health risk control programmes.

### Physicians should provide patient-centred care. (M2, PHb, PHc)

Care is said to be “patient-centred” when

- doctors focus on persons rather than diseases, and
- care is negotiated and clinical decisions agreed on with their patients, unlike in disease-centred medical practice, where doctor-patient negotiation is an implicit fact.

Patient- or person-centredness is thus a care quality criterion that prevents medicine being paternalistic, that is, patients being treated as passive objects in therapeutic relationships.

The delivery of person-centred care specifies the patient’s right to participate in therapeutic and preventive processes. It deserves to be an ethical principle because negotiating over treatment conditions the possibility of delivering eco-biopsychosocial care. To permit negotiation, the physician must be able to propose several therapeutic approaches for the same clinical problem when appropriate.

If communities and people are to adhere to public health programmes, they must also have their say (PHb). Doctor-patient negotiations on prevention are all the more necessary as it is more difficult to get people to adhere to preventive care than to secure patient compliance with prescribed treatments. This is because the personal benefits of prevention are less perceptible: avoiding suffering is delayed and benefit probability reduced, as opposed to immediate pain or fever relief in the case of an acute care prescription. In this regard, the Public Health Code correctly and pointedly provides for the right of community input in designing public health programmes (PHc).

The right to choose one’s treatment and prevention has limits. The possibility of individual choice not to adhere to a programme will depend on the patients’ knowledge/biases and their consequences for others. For instance, it is often reasonable to make some vaccinations compulsory to allow children into nurseries and day care. In addition, public health programmes should limit peoples’ ability to harm themselves. On the other hand, nutritional programmes and age limits on access to alcoholic drinks cannot be imposed on families and individuals but are legitimate.

### Information on the patient and community should be treated confidentially (M8, PHj)

The two lists agree on this principle. Clinicians and public health doctors alike should protect the confidentiality of information that could harm an individual or community if it were made public. Health promotion and education campaigns should target communities whilst refraining from disseminating information that feeds intolerance and hatred. Exceptions to the confidentiality principle must be justified by the likelihood of harming/protecting other individuals (e.g., in the case of sharing HIV status across services) and/or families. Notice that ensuring medical secrecy in a *family* medicine practice is such a challenge that some first-line services in the UK were led to abandon the use of family files. The difficulty of ensuring the anonymisation of medical data, even in public hospitals, should lead to a very restrictive policy regarding the sale of medical data by health services.

### Patient and community consent should be informed by the prevailing evidence (M7, PHf)

The medical code provides that the patient’s consent to treatment be informed by the prevailing evidence, (M7) an article that is quite parallel to that provided by the Public Health Code (PHf). Just as doctors need their patients’ consent, public health institutions should generally obtain each community’s consent to implement programmes and provide them with the information that is needed. Likewise, governments and doctors should not take advantage of the information asymmetry in their favour to sway the outcome of the process whilst biasing the mobilisation of resources (“honesty”).

### Physicians should promote the patients and communities’ participation in medical and public health services and build the public’s trust and health services’ effectiveness (M2; PHc)

WHO promoted community participation in health services as early as 1978, at the Alma Ata Conference [21]. Doctors can increase their effectiveness through initiatives promoting professional development and care accessibility and attempts to democratise them. Community participation is in turn contingent on a collaborative dialogue that brings together patients, community organisations, and health professionals.

Lengthy, sometimes frustrating, experiences in Costa Rica, Spain, and Burkina Faso show that dialogue amongst health professionals, health service users, communities, and public authorities requires publicly-oriented healthcare services [22]. This is for the following reasons:

- In commercial services, patients’ and communities’ decisions are treated as opportunity costs to which investors may object.
- Community participation in health assumes health services’ *attempting* to respond to patients’ calls for immediate alleviation of suffering and anxiety and risk mitigation. When, as is often the case in Sub-Saharan Africa and the Andean region, public health centres deliver no medical care and are restricted to implementing disease control programmes only, no real community participation can succeed. Instead, the community becomes the target of manipulation to get it to comply with the public health programme’s agenda. That is the faulty essence of social marketing applied to health programmes.

Admittedly, community participation is largely aspirational, an aspiration made necessary by the quest for effective individual and collective care and transparent public services. It is difficult to achieve and calls for long-term strategies to promote the physician’s will to engage in dialogical health education and in attempts to democratise public services.

### Physicians should respect the dignity and culture of the patient, family, and community (M1, M3; PHh)

This principle is also common to the two lists. Echoing the individual’s right to be treated with dignity and respect, public health programmes and policies ought to respect cultural differences as well. This means, for instance, that not only should Yemeni and Pakistani women be seen by female physicians and nurses; they should also be consulted, as far as this is possible, by a female carer who is curious about, understands, and respects community values and behaviours. This assumes that health professionals receive training in social anthropology and languages.

### Physicians should actively pursue equality and justice in clinical and public health practice (M6, PHd and PHl)

According to principle M6, physicians should distribute resources fairly. For instance, in using generics, physicians facilitate access to treatment by reducing its cost and thus further impact collective health. By choosing an efficient therapeutic strategy, they enable compliance with the fairness and justice principle – the possibility to render to all patients what is due to them.

Paradoxically, the public health ethical list is much less ambitious than the medical one when it comes to fairness and justice. Whilst overlooking the role of public health in sectorial fairness (e.g., avoiding inequality by disease), tenet PHd focuses the professionals’ efforts on promoting equitable health care for “disenfranchised community members”, as if medicine were for the rich and public health for the poor. Rather, we interpret the “equitable health management” principle (PHl) as an obligation for public health doctors to strive to ensure that health resources and medical care are accessible to all (i.e., leave no one behind). The objectives of such physicians’ activism need serious consideration.

Unlike some rich countries’ international cooperation agencies in LMICs, most Western Europeans would probably consider fairness in providing health care to be a central government policy of ensuring social solidarity in the healthcare sector, over and above empowering community members who would be responsible for ensuring that “the basic resources and conditions for health are accessible to all”. Admittedly, this commitment has weakened over time. For instance, the government’s share of total health expenditures on health exceeded 80% in 17 OECD countries in 1995 and in only 11 countries today.

Consequently, we propose to reconcile public health principles (d) and (l) as follows: “Physicians should treat patients and engage in forms of collaboration and affiliation in ways that build the public’s trust and health services’ effectiveness and enhance the roles of the State, regions, cities, and communities in securing equitable access to professional health care.” This means that a physician must urge her/his professional association to support fair health policies; equitable health systems; socially and professionally oriented (non-profit) health services; and ethical medical practice.

### Physicians should do no harm (non-maleficence principle) (M5; PHe)

Neo-Hippocratic ethics is based on the principle of benevolence. As such, it is essentially contrary to the central principle of neoclassical economics in that it prohibits the physician’s acting to maximise the profit/utility that s/he derives from clinical decisions.

The non-maleficence principle is mentioned in the medical list only (M5). The absence of such a tenet from the Leadership Society’s list comes as a surprise, as we see several applications of the medical principle “do no harm” (*primum non nocere*) in public health. If we bear in mind that erroneous public health decisions can cause damage on a massive scale:

- the non-maleficence tenet keeps disease control managers from hampering indviduals’ access to health care in the respective programme interventions in the available healthcare services [14].
- this principle prevents public health programme managers’ maximising health risk avoidance and, instead, reduces the programme’s impact on the health services’ resources. Except in epidemic emergencies with high case-fatality and transmission rates (as is the case with the Ebola, Covid-19, and plague epidemics), consideration for the diminishing returns of prevention should prevail in programme design: in LIC health services, for instance, very high vaccination coverage rates are generally obtained at the expense of access to other care [23].
- this principle incites manager-physicians and public health officers to promote the flexible use of the existing programmes’ medical guidelines in health services so as to avoid undermining the quality of care [24].

At the end of the day, prevention and health promotion programmes must be scientifically non-controversial to avoid harming patients, people at risk, and communities, especially because, unlike patients, their claim holders are persons with health risks only, and they did not ask anything of the health system efore being previously educated to do so.

### Physicians should engage in dual, medical/public health practice

Both clinical and public health doctors should engage in dual individual and collective health practices. Physicians should promote the public good whilst making clinical decisions and public health practitioners should treat the delivery of preventive and disease control programmes as an individual care activity and not as a mere mass intervention, as is generally the case [2]. Notice that this assumes that patients access some form of family medicine care possibly delivered, as in LMICs, by clinical officers, nurses, feldshers, or medical assistants.

### Public health physicians and clinicians should aim for professional and scientific excellence (PHk)

Whilst professional excellence is mentioned in the public health list, the medical code of ethics that we analysed does not require doctors to aim for professional excellence. This comes as a surprise, as the quest for professional excellence is a corollary of any quest for quality of care. Professionalism includes, but is more extensive than, scientific rigour. Clinical physicians must aim to be excellent professionals and not just excellent scientists for the following reasons:

- Eco-biopsychosocial clinical decision-making, negotiation with patients, and team management are a must and they largely escape scientific standardisation.
- Physicians cannot apply the recommendations of EBM blindly; they have to adapt the use of evidence based clinical guidelines to the particular conditions and values of their patients, inasmuch as guideline design is often dictated by a commercial rationale and guidelines do not lend themselves to the therapeutic management of multiple pathologies [4].
- Physicians should improve their problem-solving capacities with approaches that allow lifelong learning from their own unavoidable failures and mistakes, i.e., reflective techniques that link clinical performance evaluation to re-organising services and setting priorities in continuing medical education.
- Doctors must utilise the highest manual, behavioural, and communication skills.

In parallel, managing disease control programmes requires professional skills to address multiple political, social, and economic constraints and to enhance doctors’ problem-solving capacities and their ability to deliver quality care, especially when disease control programmes are integrated in health services. Notice that several circumstances justify not integrating epidemiological control programmes into health services: in cases of vector control; the control of diseases too rare for generalists to acquire the necessary skills; outreach to specific risk groups, e.g. commercial sex workers or drug addicts; or the control of epidemiological emergencies, for instance.

### Implementing the aetiological versus symptomatic approach to health problems (PHa) is a non-principle

Tackling the tension between addressing aetiology and symptoms is a challenge that encompasses an array of complex issues including the effectiveness, efficiency, and cost of care. Clinical and public health doctors should not systematically base solutions to individual or collective health problems on the aetiology of the disorder; the identification of a pathological agent is not always the basis for a clinical treatment or public health intervention. For instance, for the sake of efficiency, GPs, paediatricians, and disease control programmes most often do not need a differential diagnosis for diarrhoea because in children, the symptomatic treatment is generally effective without having to identify its microbiological agent.

As the above comparison shows, the medical code we propose builds on

- principles common to individual/clinical and collective/public health medicine, i.e., respect for individual and community rights,^3^ dignity, autonomy, and culture; honestly informed consent; and confidentiality of information.
- other principles with possibly incomplete equivalence need to be fleshed out: beneficence needs to be backed up by the concept of eco-biopsychosocial and patient-centred care; medical and public health activism should not just strengthen institutions, but must promote equality in health care delivery as well; and community participation ought to address health service management and community autonomy jointly.
- some stand-alone principles ought to be extended to address both medical and public health practices, namely, professional and scientific excellence and non-maleficence. Self-growth must also be an important underlying concern of professional excellence and medical education.

## Conclusion

Efforts to combine medical and public health ethics in the on-the-ground delivery of health services (not just by pronouncements of professional associations) can and will inspire a much-needed more ethical dual practice. Still, combined medical and public health ethical principles are more demanding than mere medical values, mainly because they expand the physician’s responsibility to work for collective health when in clinical practice and to deliver individual care when in public health practice. Actively advocating for public health standards in case reviews will boost doctors’ interest in professional ethics whilst becoming an intellectual challenge that calls upon doctors to question their practice in the name of their professional identity.

Ethical codes must be constantly updated, and this applies to the principles stated above. We have revised them so as to encourage discussions in all health services and medical associations. Not only are these values still largely not implemented (though partly, in progress), but they have yet to become part of a universal ambition. Avoiding ethnocentrism, we have formulated principles that address the dilemmas of modern Western medicine only, not those of Ayurvedic or Shamanic medicine, because each professional culture has its own philosophical rationale.

The implicit doctor-patient contract has increasingly been reduced to a commercial transaction, partly because most third-party payers in direct settlement schemes tend to limit their intervention to payments and profit is the motive. Adherence to neo-Hippocratic, clinical, and public health tenets as proposed here assumes that legal and payment arrangements must be implemented benevolently and compassionately, that is, with a show of respect for the dignity and human rights of patients and communities. In Deuteronomy, a combined contractual and emotional relationship is called “an alliance”, a term that could apply to the implicit clinician doctor-patient and public health doctor-community contracts discussed here.

Indeed ethical reflection does not suffice to guarantee ethical medical practice. Sufficient professional autonomy is required, and this autonomy paradoxically demands a contract and effective regulation and control. What is more, political actions are needed to make national and international health care regulation, financing, and control independent of economic forces, for in controlling health service management, economic actors impose an anti-Hippocratic, commercial rationale on medical practice. On the contrary, health services must come up with explicit strategies to enhance the morality of physicians’ behaviour. The existence of an updated code of professional conduct is a necessary but insufficient condition for their decent practice.

## Data Availability

Data sharing is not applicable to this article as no datasets were generated or analysed during the current study.

## Abbreviations

EBM: Evidence-Based Medicine
GP: General Practitioner
HMO: Health Maintenance Organization
LMIC: Low and Middle Income Countries
WONCA: World Organization of National Colleges, Academies and Academic Associations of General Practitioners/Family Physicians

## Footnotes

1 They considered the following physician’s virtues as transcending culture: fidelity to trust, compassion, phronesis (Aristotle’s practical wisdom that is, the link between the intellectual and moral life), justice, fortitude, temperance, integrity, and self-effacement.

2 The prefix “eco-” adds a dimension of individually tailored sanitation to the concept of biopsychosocial care.

3 Note that all rights pertain to individuals. Collective rights are nowadays accepted, but are not in the covenants
